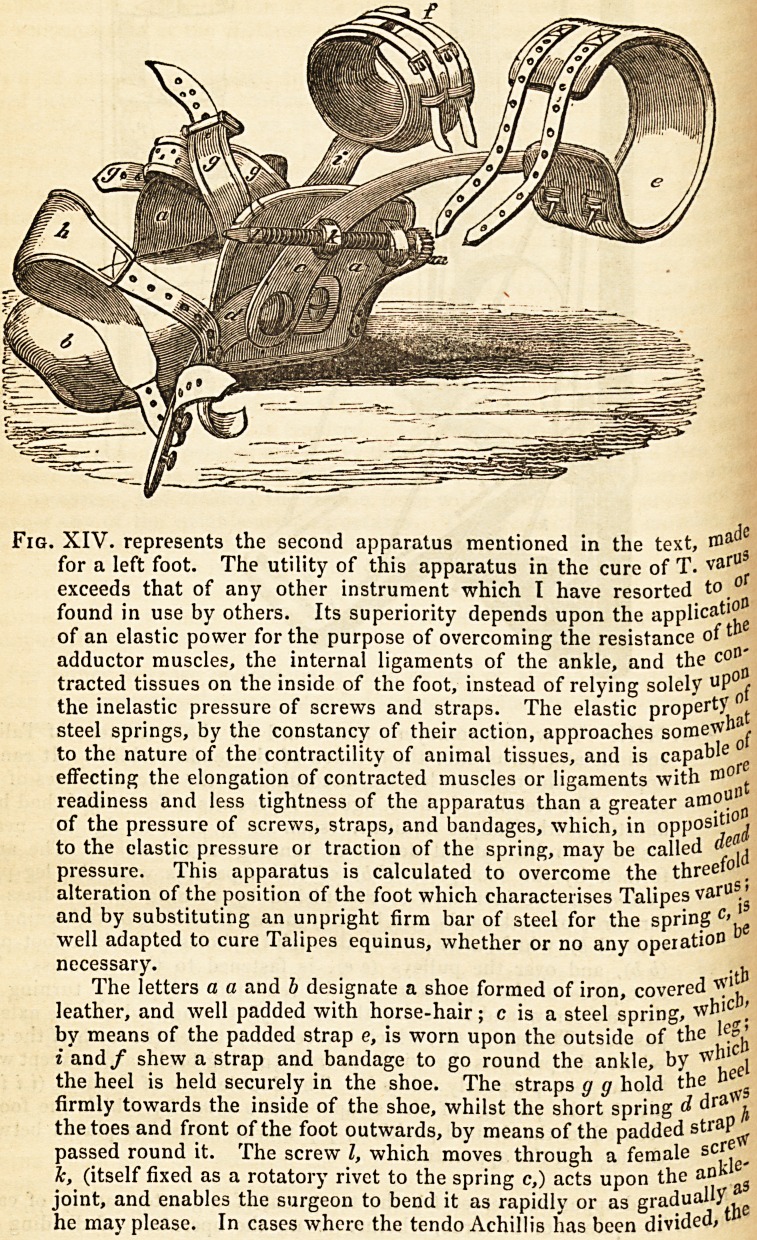# A Treatise on the Nature of Club-Foot, and Analogous Distortions ; Including Their Treatment Both with and without Surgical Operation

**Published:** 1840-04-01

**Authors:** 


A Treatise on the Nature of Club-Foot and Analogous Distortions; including their Treatment both with and without Surgical Operation. Illustrated by a Series of Cases and numerous Practical Instructions. By *W. J. Little*, M.D. Licentiate of the Royal College of Physicians; Member of the Royal College of Surgeons; Lecturer on the Practice of Medicine and on Comparative Anatomy; & Assistant Physician at the London Hospital, &c. London, W. Jeffs, 8vo. pp. 276.

Dr. Little, in his preface, states in a very perspicuous and candid manner the reasons which first induced him to investigate the nature of club-foot, and the claims of the operation of Stromeyer to success. Those reasons were of the strongest nature, for Dr. Little suffered himself from the deformity and has been relieved of it by the skill of the Hanoverian surgeon.

The volume opens with an introduction of a historical character. It displays both industry and erudition, and we recommend the curious among our readers to consult it. We shall pass at once to the operation of Stromeyer, and give an account of it, and of the varieties of malformation which require it.

The method of proceeding recommended by Stromeyer in the division of the tendo-Achillis and of other tendons is given, and we give it, in his own words.

"The operation must invariably be effected by puncture, without external incision. A very small cutting instrument should be selected—a small, moderately curved, sharp-pointed bistoury is adapted for most occasions. The limb should be extended, in order to produce the necessary projection of the tendon, when the instrument should be passed behind it, the point perforating the opposite skin; division of the tense resisting tendon being effected rather by pressure of the edge than by its slow and cautious onward movement. The skin being elastic yields to the pressure of the knife, the two punctures not exceeding its width. I have frequently divided the tendo Achillis in this manner without producing a second puncture; but this is of little moment, as two minute punctures heal as quickly as a single one. The division of the tendon is known by an accompanying sound, which can scarcely be mistaken. The performance of the operation with the point of the instrument is less to be relied on, partly from its being too weak, and also because the operator can be less certain of not causing injury to other structures in the event of the patient not remaining quiet throughout the operation..... The attempt to commence extension directly after the operation, and the endeavour immediately to restore the limb to its natural position, which will very seldom succeed, and, as the case of Sartorius proves, can only be effected by great force, is neither necessary nor advisable. The commencement of extension before cicatrization of the wound in the integuments is unadvisable even when possible, as it may produce inflammation and suppuration not confined to the vicinity of the wound; it is unnecessary, inasmuch as the tension of the divided muscle is not restored during the gradual mechanical extension applied subsequently to the healing of the wound, but occurs after the complete reunion of the tendon and after the necessary motions of the limb during exercise have acted as a stimulus to its contractility ..... The alteration in my method proposed by Bouvier as an improvement, viz., the replacement of the foot immediately after the operation, must, with the greater justice, be regarded as a retrograde progress, from its having constituted the plan of my predecessors." lix.

Stromeyer observes that the assertion of M. Bouvier and Mr. Whipple of Plymouth, that the extension is more painful when effected after the healing of the wound, is entirely imaginary; as the pain so produced is never referred by the patient to the divided part, but to other tissues still resisting the extension—the tibialis posticus and peronei muscles, and the posterior ligaments of the joint.

Dr. Little thinks that little real improvement has been effected in the operation since Stromeyer improved on the previous one of Delpech. Dr. Little adds, *that* the position of the patient, whether prostrate or sitting, may depend on the convenience or fancy of the surgeon—*that* the best spot for dividing the tendo-Achillis may be either an inch above the extremity of the os calcis or where the tendon is most prominent—and *that* the form of the small knife employed matters little, provided the surgeon divide the tendon by puncture, with little injury to the surrounding parts.

"The most simple, secure, and certain mode of severing the tendo Achillis, is first to apply the edge of the knife to the anterior surface of the tendon, cutting from the deeper-seated tissues towards the integuments. The proposition to divide the tendon in an oblique direction, in order that larger surfaces may be presented for the effusion of the uniting medium, and firmer reunion of the tendon be ensured, has resulted rather from mechanical ideas of the mode of reunion of divided tendons than from practical observation or physiological reasoning. I have invariably performed the transverse division, and in every instance the tendo Achillis has united by a band as firm and large, and sometimes even larger, than the original tendon; the spot where the operation has been performed is frequently imperceptible The experiments of Von Ammon, Günther, and Bouvier, on dogs and horses, have demonstrated that reunion of tendons, by a strong ligamentous or semi-cartilaginous band, takes place, in whatever manner the division be effected, and whether the ends be maintained in apposition, or at a distance, resulting from contraction of the muscle, or even when portions are removed." lxi.

Dr. Little goes on to remark that the principal improvements have been made in the apparatus—in the more complete definition of the anatomy, pathology, and varieties of Talipes—and in the demonstration of the certainty of cure in a larger proportion of the severer forms of distortion. And Dr. L. closes his Introduction with a well-merited encomium on Stromeyer.

We quit this portion of the volume, and proceed to that which treats of the varieties of club-foot, their causes, and their management.

Dr. Little remarks that the term club-foot has been indiscriminately applied to three kinds of deformity, to which surgical writers have affixed the terms *varus, valgus, and pes equinus;* and the ordinary laconic description of their symptoms has not usually extended further than stating, that in varus the toe of the affected limb is twisted inwards, so that the patient walks more or less upon the outer ankle; that valgus is the contrary deformity, the toe being turned outwards, and the patient compelled to tread upon the inner ankle: and that the pes equinus is that state of the foot in which the individual rests the weight of the body upon the toes only.

But, he continues, as surgical means of overcoming these deformities have been discovered, a more strict description of their symptoms is necessary to distinguish the cases which are fitted for operation from those which are curable without an operation, or from others which are incurable by any means.

"The nomenclature, for the same reason, has appeared to me to require some additions; the causes and varieties of deformities of the feet being numerous, the three names, varus, valgus, and equinus, are insufficient to designate them. I have therefore proposed to employ the classical word *Talipes* (hitherto applied to one species only), as a generic term, to include all those deformities of the feet produced, as will be seen, by contraction of certain muscles, to restrict it to deformities from this source; and to use the terms varus, valgus, and equinus, to designate the specific forms of these diseases." 2.

1. The most simple of these deformities is the Pes Equinus, or Talipes Equinus. It is that contraction of the limb, where the individual walks solely upon the point of the foot, upon the toes, or upon the ball of the foot; without the heel or any of the posterior part of the sole touching the earth. The heel may be more or less elevated from the ground, from half an inch to five or six inches, according to the different degrees of severity of the disease. There is also, adds our author, great variety in the manner in which the individual treads, as the weight of the body may be borne either upon the front part of all the metatarsal bones, which constitutes a pure case of Talipes equinus; or the person is inclined to tread more upon the little toe, or upon the great toe, when the deformity approaches the other forms of disease hereafter to be considered. Most frequently in such cases the ball of the little toe bears the brunt of the pressure of walking, from the toes becoming thrust a little inwards.

Dr. Little subjoins some woodcuts, illustrative of the Talipes Equinus.*

1. *Congenital Talipes Equinus in a youth aged* 14 *years.*

The deformity of this foot, and of every T. equinus figured in this work, appeared much *greater* in the act of walking than represented in the drawings, owing to the toes then turning a little inwards, and from the individual wearing a high-heeled boot, having a clumsy appearance.

* We are indebted to his kindness for the opportunity of presenting these and other woodcuts to our readers.

2. *Non-Congenital Talipes Equinus in a youth aged* 11.

2. Dr. Little proceeds to state that the most common form of club-foot is the TALIPES VARUS—that in which the patient is said to walk upon the

Fig. 3.—*Example of congenital T. varus affecting both the feet of a child aged* 20 *months.*

outer ankle, the toe being turned inwards. It is that deformity in which the foot from some cause undergoes a threefold alteration of its position in relation to the leg—extension, adduction, and a rotation of the foot, somewhat analogous to supination of the hand, taking place to a greater or less extent according to the severity of the disease. The heel is drawn upwards (extension), the toe is turned inwards (adduction), and the patient treads upon the outer edge of the foot only, the inner edge being raised from the ground (rotation). This threefold alteration from the natural position of the foot occasions the most serious impediment to steady or comfortable walking; and, when the disease reaches the higher gradations, the foot assumes a frightfully distorted appearance.—(See figs. 5 and 6.)

Fig. V.—*Front view of an adult congenital T. varus.* Shewing the deformity of the tarsus and the swelling (*b*) which result from the attrition to which the back of the instep is subjected; *h*, the heel; *m*, the malleolus externus. A similar deformity of both feet existed in the subject from which the drawing is taken.

FIG. VI. *represents the same foot as fig.* 5, *in the reverse position.* In this example of the highest grade of deformity, the sole of the foot is completely vertical, instead of horizontal. The letter *h* indicates that surface of the heel which in the natural state of the foot would touch the ground, whereas it is here presented directly backwards; *m* is the malleolus externus; *b*, the tumor formed of successive layers of thickened cuticle, cutis, and thickened adipose, ligamentous, and periosteal tissues, constituting a cushion on which the patient walked. The outline of this tumor is seen to be better defined upon the posterior aspect of the foot than upon the anterior aspect.

3. VALGUS, OR TALIPES VALGUS, is a much more rare affection than either of the preceding. It may be regarded as the opposite state to T. varus, and, like it, consists of a threefold alteration of the position of the foot, there being partial bending of the ankle, *ab*duction, and a rotation of the foot; but this rotation takes place in the opposite direction to that in Talipes varus, as in T. valgus the external *edge* of the foot is removed from the ground. The rotation in a complete case of T. valgus is so great, that the patient in the act of walking does not touch the ground with any part of the sole of the foot, but treads entirely upon the inside of the instep and upon the malleolus internus. In short, the sole of the foot is directed completely outwards and a little backwards, the ankle is held in a state of semi-flexion, the anterior half of the foot (the metatarsus and toes) not touching the ground.

Fig. IV.—*Front view of a congenital Talipes valgus of the right foot of a boy aged four years.*

*d a*, the outer edge of the foot raised from the ground.
*e*, the great toe raised from the ground, although the internal edge of the foot is directed towards it.
*b*, the internal malleolus; and *c*, the internal extremity of the navicular bone; being the parts upon which the patient walked.

Dr. Little remarks that, in each of the three preceding forms of disease, the surgeon may, in cases not of too long standing, reduce the foot temporarily with his hands, and ascertain the obstacles to its natural position. In the Talipes equinus and T. varus, he will feel the tense tendo Achillis resisting his efforts to bend the ankle-joint; in the T. varus, he will, according to the stage of the disease and the state of the limb as respects the quantity of its adipose tissue, feel, and occasionally see, the contracted tendons of the tibialis anticus and tibialis posticus muscles; in both diseases he will often notice the contracted state of the whole of the muscles of the sole of the foot, and of the tendon of the long flexor muscle of the great toe; as well as the elongated condition of the tendons of the muscles on the front of the leg. The toes, especially the great, are held by them in a state of semi-extension.

"The tendons which in Talipes valgus are felt to obstruct the restoration of the foot to its natural position, are, as far as my experience has hitherto extended, chiefly those of the peronei muscles. In a case of congenital T. valgus at the age of four years (Fig. 4), which I have recently operated with success, but only the second case of true Talipes valgus which has been presented to my observation during the last two years, although more than a hundred instances of the other two forms of Talipes have passed through my hands within the same period,—the tendons of the contracted peroneus longus and brevis muscles had been felt very prominent from the time of birth. Owing to the alteration which had been effected in the relative position of the bones which enter into the composition of the ankle-joint and tarsus, the tendo Achillis deviated outwards from its usual direction, similar to the tibialis anticus in some cases of T. varus, offered a strong resistance to replacing the foot, and was consequently divided." 6.

The general condition of the limbs is similar in the three forms of Talipes. But it differs from that of a sound limb, the legs being proportionately shorter and the feet smaller. When one limb only is affected, the difference in the length of that and of the sound one varies from a quarter of an inch to four. The bones in these cases are generally shorter, and more slender than in a natural foot. The thigh occasionally participates in the shortening, and its muscles are invariably weaker in the case of a single Talipes than are those of the sound side. The muscles of the calf and front of the leg are often not one third the usual size.

The mode of progression, continues Dr. Little, necessarily adopted by those persons afflicted with the various forms of club-foot, is not only disagreeable to the eye of the casual observer, but often painful and extremely laborious, even when only one limb is affected. By dint of long practice many are enabled to balance their bodies tolerably well in walking, although the base upon which the weight of the body is borne is so much smaller than that afforded in the natural state by the *soles* of the feet. In order to compensate for deficiency in this respect, they usually stand with the extremities widely separated, and use the arms as a means of balancing the trunk. Few, however, can take long walks, partly on account of the exertion, partly by reason of the pain. Bursæ, corns, &c. form, as we might anticipate.

Contraction of the knee is not unfrequently induced by the continued operation and contraction of the gastrocnemius. In the majority of cases of Talipes in which the knee is bent while the patient is in the erect posture, it can be straightened when he is seated, as the stress maintained upon the gastrocnemii when the weight of the body is thrown upon the toes is then removed. But on attempting to straighten the knee when erect the elevation of the heel is proportionally increased. Dr. L. has, however, treated a case where, not only was the heel drawn up several inches, but the knee was kept bent at a considerable angle in every position of the body.

Another complication, he adds, which occurs in Talipes, is an unnatural inward or outward rotation of the thigh, and consequently of the entire extremity. This arises from an inequality in the power of the adductor and rotator muscles of the thigh, and is probably an affection of those muscles analogous to that of the muscles of the leg implicated in Talipes.

*Morbid Anatomy of Talipes.*—Dr. Little has made numerous dissections, for the purpose of elucidating the actual condition of the parts in Talipes. Hitherto little had been done in that way.

In *Talipes Equinus* there does not seem to be, nor is there, any great deviation from the natural form and arrangement of the tarsal bones. The following is a representation of the bones of a foot affected with Talipes Equinus, and contained in the Museum of the London Hospital.

*The preparation of T. equinus alluded to in the text. It will be observed that the toes are not extended as in Figs. 1 and 2. This depended, probably, on the subject of the preparation never having placed the foot to the ground, as he had walked on the knee, with the assistance of a wooden leg. It happens occasionally, however, in T. equinus, when the anterior extremities of the metatarsal bones are drawn to a great extent towards the under part of the os calcis, that the individual walks on the dorsal surface of the metatarsus and toes instead of on their plantar surface.*

It will be observed that their development is imperfect, but there is no positive displacement of any particular bone. Owing, remarks our author, to the extended position of the foot, the surfaces of the astragalus which should articulate with the tibia and fibula are exposed upon the dorsum pedis, and, as well as the articulating surfaces of the tibia and fibula, have lost much of their natural smoothness in consequence of little or no motion having taken place between them. The os calcis, during life-time had been drawn so much upwards behind the tibia as to have touched it; owing to which a new articular surface is visible upon the superior part of the calcaneum, close to the articular surface for the astragalus. The dorsum pedis is much more convex than in a natural foot, owing to the scaphoid, together with the cuboid, cuneiform, and metatarsal bones being drawn towards the sole; hence the round head of the astragalus is exposed, instead of being covered with the hollow of the scaphoid bone, as in a healthy state.

*Talipes Varus.*

Dr. Little has found the alteration in the position of the bones more complicated in this than in Talipus equinus. It presents all the anatomical characters of the latter, with the addition of such as depend on the adduction and rotation of the foot. The os calcis is drawn upwards; the tibial articular facets of the astragalus and its round head are exposed upon the dorsum of the foot; but the scaphoid, cuboid, cuneiform, and metatarsal bones are not merely drawn towards the sole, but also inwards and upwards, so that the innermost point of the navicular bone occasionally touches the internal malleolus, and has an articular surface formed on it, occasioned by constant friction. The superior or external surface of the os cuboides is somewhat separated from that of the os calcis; whereas the plantar surfaces of these two bones are turned towards each other, leaving a triangular space between them externally. The course of the tendons of the muscles situated upon the front of the leg is consequently much altered. Those of the tibialis anticus, extensor proprius pollicis, and ext. long. digitorum, more particularly the first of these, are deflected so much internally, after passing over the ankle-joint, as to serve by their action to increase the deformity in the living subject.

The accompanying diagram represents the condition of the bones and tendons in a foot affected with Talipes varus. It is that of an adult female.

It may be relied on in the study of the anatomical characters of this disease; as, from the evaporation of the spirits of wine from the jar in which it had been preserved undissected, it was completely dried up, resembling the foot of a mummy. Owing to this unexpected but fortunate circumstance, I was enabled (by soaking the external part of the foot in warm water, and by subsequently removing as much of the shrivelled skin and fascia as was necessary,) to expose the bones for examination without the slightest alteration of the relative position of those of the tarsus having taken place since death.

*Talipes Valgus.*—Dr. Little has enjoyed only three opportunities of investigating the state of the parts in this.

"The subjects from which the preparations were taken were full-grown fœtuses. In these, so far as the incomplete development of the osseous structures enabled me to judge, the astragalus was twisted in such a manner, that the articular facet which ought to be applied against the inside of the internal malleolus did not enter the composition of the ankle-joint, but was turned downwards; the navicular bone and calcaneum followed the astragalus, and, together with the internal malleolus, would have touched the ground by their internal surfaces, had the feet belonged to subjects who could have walked. The external edge of the os cuboides and fifth metatarsal bone and external surface of the calcaneum presented directly upwards; the latter, therefore, was in contact with the external malleolus, the prominence of which could not be felt through the skin of the foot." 15.

Dr. Little passes to the *Causes* which produce, or which maintain the deformity of Club-foot. He contents himself with stating the results of the researches of Rudolphi, Sörg, Stromeyer, and himself.

1. He first alludes to the popular (medical as well as vulgar) idea, that it is a monstrosity, a lusus naturæ, &c. and he touches on the notion which would ascribe it to a fright, a view of some similar deformity, on the mother's part, while pregnant. He reasonably attaches small faith to this.

2. "External injury to the limbs during fœtal existence—such as pressure by the parietes of the womb, or the limbs becoming entangled, and compresing each other,—is the next cause more generally admitted as the source of these deformities. But I must briefly dismiss the consideration of this alleged cause. It will suffice to inquire—even if the relatively large quantity of liquor amnii in which the fœtus is submerged did not, at the early period of uterine existence at which these deformities are to be found, oppose an effectual obstacle to the exercise of pressure upon any portion of the fœtus apart from the remainder,—how it is possible that pressure of the walls of the uterus—a mechanical cause acting from without, should affect the fœtal limbs, by disturbing the arrangement and development of certain bones and muscles according to certain invariable rules, instead of acting in an uncertain manner, as external mechanical agents always act when brought in collision with the living body." 17.

3. The circumstance, he says, which throws most light upon the nature and cause of club-foot, combined with a knowledge of the morbid anatomy of the disease, is, that it very often takes place after birth and at various ages—most frequently during childhood, from the age of a few months to three or four years; that in these non-congenital cases it presents the same inflexible and essential characters as the congenital affection.

A child, he continues, during the progress of dentition, is observed "slightly to drag one leg," or may have a little limp in his gait, depending, although the cause may be overlooked, on a slight rigidity or contraction of the muscles of the calf, and consequent stiffness or inability of completely bending the ankle-joint. The former case arises from partial paralysis of some of the muscles of the leg, usually those situated on the front, denominated flexors of the ankle-joint; the latter from inordinate or spasmodic contraction of other muscles, usually those on the back of the leg, the so-called extensors of the foot. The disease, from whichever of these two causes it may arise, advances unchecked by *medical* treatment, and the result is the same, the heel being permanently raised from the ground, constituting Talipes equinus; or the toe being drawn inwards at the same time, producing Talipes varus.

In other cases, a child exhausted by illness, or during an attack of spasmodic croup has "a fit," which is succeeded by the loss of one or of all his limbs. This loss of the use of the limbs is of two kinds—*paralytic*, in which case it is very often paralysis of some or all of the muscles of one side, but most commonly paralysis of one or both of the lower extremities; or it arises from the seizure of some of the muscles of the extremities with involuntary, tonic contraction—*spasmodic* loss of the use of the limbs. Again, this spasmodic affection of the muscles may affect even the trunk, in addition to the limbs, giving rise to deformity of the spine. Sometimes the loss of the use of the limbs has occurred during the comatose stage of fever; and on recovery, the inability to stand or walk is very reasonably attributed to general debility. But whilst "strength" may be recovered in every other part of the frame, the patient continues unable to place the heel or heels upon the ground (T. equinus), or that symptom is conjoined with a turning inwards of the toe or toes (T. varus). Dr. Little has seen one and only one instance of Talipes valgus, occurring under such circumstances.

"The lameness, whether arising from paralysis of some of the muscles, or from the involuntary contraction of others, is usually accompanied with a diminution of the entire vital energies of the limb, which proves that the organic system of nerves has also suffered—its temperature is often below the natural standard; the muscles either waste, or, with the bones, cease to grow as rapidly as those of a sound limb; the ligaments even do not evince their proper firmness—for instance, the knee, provided none of the muscles of the thigh be affected with spasm, admits of more lateral motion than in a natural state. Talipes is not only produced in persons of a tender age, but likewise at a later period of life, and even after the age of puberty, in those whose nervous system is equally susceptible of disease. I have witnessed the formation of perfect cases of club-foot,* through spasm of the gastrocnemii and anterior and posterior tibial muscles, in hysterical young women, who had previously enjoyed the perfect use of their limbs." 21.

Dr. Little examines, a little more in detail, these non-congenital contractions of the feet. He takes, *first*, a case originating from paralysis of the anterior tibial muscles. Some lesion of the nervous centre in communication with their nerves, induces the paralysis in question. The posterior muscles of the leg (those of the calf in particular) having lost their natural antagonists, become firmly and permanently contracted, by the constant action of their involuntary contractile power, by which the heel is raised from the ground, constituting Talipes equinus. At an early period of the disease, this contraction may be overcome by forcibly bending the foot with the hand; but this after a time becomes impossible.

---

* "Can so unclassical a term be applied to a disease of a limb occurring in a young adult female? and if not, need we, in order to designate the congenital cases, longer use a term which persons of nearly every class appear disposed to repudiate?"

"The other case, that of a Talipes originating from spasm, admits of a different explanation. The remote cause resides either in the central organs of the nervous system (most probably in the spinal marrow), or it is a disease existing in some other organ of the body, affecting peripheral parts of the nervous system; for instance, in some one of the viscera of the chest or abdomen, more probably of the latter. From this an injury is propagated to the central organ, and is reflected to certain muscles of the leg, which become spasmodically contracted. In other words, there may be either some deviation from the healthy state in a part of the spinal marrow, where the roots of the motor nerves distributed to the muscles of the calf are implicated or irritated, causing them to become involuntarily contracted; or there may exist elsewhere some disease, such as an irritation of the mucous membrane of the alimentary canal by improper or undigested food or worms, through which filaments of nerves (named by Dr. Hall *incident)* are excited. These communicate in the spinal cord with other filaments—the *reflex,* or involuntary motor nerves, whereby the muscles of the calf are excited to spasmodic action." 22.

In the Talipes varus, the paralysis or spasm affects a larger number of muscles. When paralysis is the cause, the peronei muscles have lost their power, as well as the anterior tibial and long extensor muscles of the toes. If spasmodic contraction be the cause, the posterior tibial muscle, long flexor muscle of the great toe, those of the sole of the foot, and sometimes the tibialis anticus muscle, are partially involved in the production of the deformity.

Contraction of some of the muscles of the arms occurs, after birth, not unfrequently, and arises from the same causes as have been enumerated. Dr. L. has met with two cases unaccompanied with pedal contraction, and he has treated several in which they were conjoined.

Other causes besides those enumerated, which produce a shortening of the muscles and other soft parts upon one side of the leg, by disturbing, although in a different manner, the antagonism of the muscles, are capable of producing deformities similar to those belonging to the genus Talipes. Dr. Little has had under his care two examples of the ankle-joint rigidly fixed in the extended position; in one instance the heel having been raised three inches, and in the other from five to six inches, in consequence of the contraction of numerous cicatrices on the back of the leg, resulting from the healing of abscesses, accompanied with loss of substance through sloughing and necrosis. Another cause of the shortening of the muscles on the back of the leg arises from the long confinement of patients to bed, during the union of some kinds of fractures, or during the reparation of other injuries. Dr. L. has also met mith a case of permanent elevation of the heel produced by the ankle-joint having been too tightly secured by mechanical apparatus, and continued too long in the extended position, for the union of a ruptured tendo Achillis. The contraction had also been increased by the patient having worn high-heeled shoes. Dr. Little quotes an observation of Dr. Stromeyer's, *that* rupture of the tendo Achillis is an accident too much meddled with. He deems the application of the slightest bandage, for the first few days, sufficient. We are inclined to believe this is falling into an extreme laxity. The cases that we have seen of ruptured tendo-Achillis* have proved rather troublesome.

* Rupture of some fibres of the Gastrocnemius is more common.—Eds.

"Having thus offered my opinions of the causes of those deformities of the feet which take place after birth, and stated the identity of their symptoms and morbid anatomy with those of the club-foot with which children are born, the probability will, I think, appear obvious that the remote causes are the same: but there are other phenomena connected with the history of these affections which render the accuracy of these opinions almost capable of demonstration. Fœtuses which have suffered some evident derangement in the development of the nervous system, such as those denominated hemicephalous and acephalous, or affected with spina bifida, and those born before the expiration of the natural period of utero-gestation, are particularly obnoxious to this deformity of the feet. The occurrence of the perfectly analogous deformity of the hands which takes place prior to birth, denominated club-hand, in which the flexors and pronators (analogous to the so-called extensors and adductors of the foot) are likewise contracted, corroborates the opinion that congenital club-foot depends on spasmodic musular contraction. In the instances which I have examined, of congenital deformity of the hand (club-hand), both in museums and in the living subject, the feet were also affected with Talipes, proving the operation of a common cause. Other circumstances corroborative of this opinion, are the co-existence with congenital club-foot of congenital squinting, and even congenital stammering or mis-enunciation,—diseases which evidently depend either on the increase of the involuntary or the decrease of the voluntary motor powers of the orbital and laryngeal muscles.

The importance of these facts is increased by the observation I have made, that non-congenital club-foot is likewise occasionally accompanied with strabismus." 25.

Before we pass to the treatment of Talipes, we must introduce, from the Appendix to the present work, a notice of another form of the disease, which our author has designated *Talipes Calcaneus*. What account he furnishes, is contained in the history of a case.

*Case.*—Nov. 1838. Miss * *, aged four and a half years, is stated to have been born with a perfect condition of the feet, but was observed, soon after learning to walk, to have acquired considerable lameness of the left foot, apparently from weakness. The thigh and leg decreased in thickness, the muscles became lax, and the temperature diminished. She gradually discontinued treading on the entire sole, the heel principally receiving the weight of the body, and the front part of the foot becoming elevated. At present, when the patient is seated and the limb is at rest, the foot is distorted in the manner represented fig. 39, its anterior part being elevated to the utmost extent, from contraction of the anterior tibial and extensor muscles of the toes, the long axis of the os calcis being situated perpendicularly beneath the axis of the leg, and the posterior surface of the heel touching the ground instead of its inferior surface: in short, the foot is involuntarily flexed to the fullest extent. No inequality in the elevation of either edge of the foot exists. The tendons of the anterior muscles of the leg are felt tense and prominent; the tendo Achillis, on the contrary, can with difficulty be discovered, so flattened and attenuated has it become from the constant elongation to which it has been subjected; its relation to the deeper-seated muscles is also considerably changed. Instead of the existence of a considerable space between these muscles and the tendo Achillis, the latter extends like a thin tape towards its insertion, being closely applied to these muscles and the posterior surface of the ankle-joint—the result of the os calcis being placed almost perpendicularly beneath the tibia, instead of horizontally or obliquely.

FIG. 39.—*Talipes calcaneus in a child aged four years and a half.*

Dr. Little observes, in reference to the treatment of this case:—

"As the patient was able to walk without any considerable difficulty, weakness and an occasional tendency to fall being the chief inconvenience of which she complained, and the foot being by pressure easily reducible to the proper form, indicating the absence of structural shortening of the anterior muscles of the leg, I did not consider division of their tendons advisable, but recommended the wearing of a boot furnished with a thin steel support on one side, so constructed as to maintain the foot bent to a right angle only, and cause her to tread equally on the entire sole." 264.

The boot has completely answered the purpose intended, as she treads equally on the entire sole, and walks without the appearance of lameness. The application at bed-time of a tin splint, constructed so as to maintain the foot in the extended position, has been recommended, in order to prevent contraction of the anterior muscles of the leg during the night.

We now proceed to the—

### *Treatment of Talipes.*

Dr. Little does not propose to embrace all the minor and more circumstantial features of management, but he states the principles to be followed in every case.

Hitherto, he observes, the deformity has been too often regarded as simply a physical alteration of certain parts of the limb; certain tendons or muscles have merely been considered as too *short*, and the bones of the tarsus deformed, or, in the worst cases, unnaturally jumbled together: consequently a mechanical plan of treatment best accorded with these ideas of the pathology of Talipes, it being considered that no contracted living tissue, such as a wasted muscle or "*shortened tendon*," could long resist the operation of a well-applied mechanical power, properly adapted to elongate the contracted parts. Experience has proved this opinion to be erroneous. Each case must be treated in reference to its nature, and the nature of each must form a special subject of investigation.

Thus, if Talipes originates after birth in paralysis of the anterior muscles of the leg, the surgeon should treat that on the same principles as paralysis of any other part of the body. If the case proceed from active permanent, and involuntary contraction of the muscles of the calf, or the other muscles of the foot, producing Talipes equinus or T. varus, he will, as in the treatment of paralysis, direct his attention to the central organs of the nervous system, the brain and spinal cord, and search for the remote disturbances in the chylopoietic or other viscera. Mechanical means are, at the same time, necessary to prevent the muscles from becoming affected with structural or organic shortening, and manipulation, friction, and proper apparatus are to be employed.

"Even a congenital case, where the contracted muscles may be elongated by the hands, and the foot thus placed nearly in its natural form, should from analogy be treated in the same manner. I have treated a child who had never walked (of the age of two years and a half when first placed under my care), born with spasm of some of the muscles of the eyes, of the spine, of the adductors of the thighs and muscles of the calf, producing squinting, partial opisthotonos, rotation inwards of the thighs, and double Talipes equinus. The whole of these affections, with the exception of the strabismus, have been almost entirely removed by the use of ferri sesquioxydum, laxatives of hydr. c̄. cretâ, with pulv. rhei. and long-continued extensive counter-irritation to the spine, with the addition of frictions and manipulations." 27.

There are many cases both of congenital and non-congenital Talipes, in which medicine is unnecessary, either from subsidence or hopeless inveteracy of the paralysis or spasm, and where there remains only an inconsiderable *structural* shortening, although accompanied with great deformity. These cases may be cured in the space of a few months, without relapse, by means of mechanical treatment alone.

"If, however, either in congenital cases, in consequence of the affection of the muscles having occurred at an early period of fœtal existence; or in non-congenital cases, from the deformity having continued unchecked for too great length of time,—structural shortening of the contracted muscles have taken place, which is usually ascertained by the inelastic rigid nature of the contraction of the muscles, the tendons of those muscles which resist the restoration of the foot to its proper form, must, provided that there be no accidental displacement or deformity of the bones, or ankylosis, be divided by the knife, in order to obtain a cure.

In most cases of Talipes equinus, and in many of T. varus, which require an operation, the tendo Achillis only is required to be divided; in other cases of Talipes equinus I have sometimes found it necessary likewise to divide the tendons of the tibialis posticus and flexor longus pollicis muscles. In severe long-standing cases of Talipes varus the section of the tendons of the anterior tibial, posterior tibial, extensor proprius and flexor longus pollicis muscles is requisite, in addition to division of the tendo Achillis, in order to facilitate the restoration of the foot to its natural shape and position. The case of congenital Talipes valgus already mentioned will indicate the parts which it may be found necessary to divide in that form of disease." 28.

Dr. Little insists on the principles laid down by Stromeyer, in reference to the operation. Those principles are as follows:—

The tendons of the muscles which maintain the deformity should be divided with as little injury as possible to the skin and neighbouring parts.

No attempt should be made to force the foot into its natural shape immediately after the operation; but the necessary extension for that purpose should be commenced as soon as the external puncture or punctures are completely healed: this occurs about the second or third day.

The lymph which is effused between the ends of the divided tendon or tendons, with the muscles that are not divided, and the ligaments and fasciæ which may impede the replacement of the foot, must be gradually extended until the foot assumes its natural shape, and the ankle can be bent to its fullest extent.

The application of the apparatus by which extension is effected must be continued for a certain period after the cure, notwithstanding that the patient has been enabled to stand firmly, and has improved in walking, in order to obviate the tendency to contraction evinced by the intermediate substance or lymph effused between the ends of the divided tendon.

Dr. Little states, with justice, that the safety of the operation hinges essentially on the small size of the puncture, and the speedy healing of the wound. Dr. Little has used a small, curved, sharp-pointed bistoury, having a concave edge, the cutting part of the blade seven-tenths of an inch in length, and the greatest width one-tenth of an inch. On the whole, however, he finds that a knife with a straight or slightly convex edge is preferable to one too much curved or too concave: but, provided the operator possess ordinary dexterity, the form of the knife which he uses will be of but little consequence.

He performs the operation thus:—

The patient being seated, an assistant supports the knee, whilst another, drawing downwards the patient's heel with his left hand, and pressing upwards the toes and front of the foot with his right, produces the necessary tension in the tendon proposed to be divided. The operator, after feeling the outline of the tendon with the left fore-finger and thumb, passes the bistoury through the skin, one or two fingers' breadth above the malleolus internus, with one of its sides turned towards the tendon, and the other directed towards the deeper muscles and the tibial vessels and nerves. On being satisfied that the point of the knife has been passed beyond the external edge of the tendon, and has nearly reached the skin of the opposite side, the knife is turned so as to bring the cutting-edge to press against the anterior surface of the tendon, which is then divided by the action of withdrawing the knife from the limb, and commonly by a single stroke. The complete division of the tendon is known by the immediate cessation of the tense resistance, by hearing a distinct snap, and by feeling, before the knife is wholly withdrawn, that nothing remains undivided except the flaccid integuments. The operation does not occupy a quarter of a minute, and is almost bloodless, as usually not more than a single drop of blood is effused.

" The division of the tendon of the posterior tibial muscle is, in my opinion, best accomplished at the distance of two or three fingers' breadth above and behind the internal malleolus. The point of a strong and straight bistoury should be introduced through the skin at the outer edge of the tendon, and passed between it and the tendon of the long flexor of the great toe, directed towards the tibia. As soon as the knife reaches the bone, the handle should be depressed outwardly, and the point carried internally beneath the posterior tibial tendon, and continued onwards until the surgeon is satisfied that the point has passed beyond the inner edge of the tendon. He may then feel that he has the tendon upon the edge of the knife, when by a few slight cutting motions he may divide it without difficulty. No snapping sound, similar to that which follows the division of the tendo Achillis, is heard when the section of the posterior tibial tendon is accomplished; as the fleshy fibres of this muscle take their origin so low towards the malleolus internus, that they prevent the occurrence of any considerable retraction of the superior end of the tendon.

The most favourable situations for dividing the tendons of the tibialis anticus and flexor longus pollicis muscles, are where the former passes in front of the ankle-joint, and where the latter is felt most prominently in the sole of the foot, in those cases where division is required. The manner of dividing each of these tendons is to pass the point of a bistoury through the integuments, and then with great care beneath the tendon, avoiding to carry the knife farther than is absolutely necessary, and dividing the tendon from within outwards, in order not to endanger any of the neighbouring structures. The recoil of these muscles, on their tendons being divided, is distinctly felt and heard. If they be thus cautiously divided, no risk is incurred of injuring the anterior tibial, posterior tibial, and internal plantar arteries, or any of the nerves. The wounds made in the integuments are extremely small, and unite by adhesion; consequently all chance of suppuration and sloughing is avoided." 31.

After either of these operations, a strip or two of sticking-plaister, and a wooden or a pasteboard splint, intended to keep the foot in its *deformed* position, are to be applied.

" The apparatus to which I resort for the necessary extension of the limb, is the foot-board invented by Stromeyer (fig. 13), and another apparatus (fig. 14), constructed in some respects upon the principle of that described and represented by Scarpa in his work upon the cure of these deformities. The original instrument of Scarpa, although highly valuable in affording one of the principles upon which a correctly adjusted and efficacious instrument for the cure of these contractures must be made, was entirely defective in providing means for drawing the heel downwards. This Scarpa anticipated would gradually take place when the patient walked, the tendency of the toe to turn inwards being at the same time obviated, and the foot maintained securely in a favourable position by his apparatus. Stromeyer added several improvements to Scarpa's instrument, from which, in addition to my experience and numerous private suggestions received from him, I have profited, by a different adaptation of the screw to act upon the ankle-joint, and by altering the arrangement of the straps. The mode of applying Stromeyer's foot-board and the latter apparatus will be understood by an examination of the figures. The former is most useful in the treatment of Talipes equinus, although it may be resorted to in those cases of T. varus which have not attained the more advanced stages of the deformity. Its especial utility in T. varus arises from the very ingenious contrivance of the foot-part, which may be rendered more or less oblique, and thus accommodated to the obliquity of the sole of the deformed foot.

FIG. XIII.—View of Stromeyer's foot-board for the treatment of Talipes equinus and varus after the division of the tendo Achillis. It can be applied to the right or left leg, according to the circumstances of the case. The smaller of these figures represents a foot which had been affected with Talipes equinus, (such as is seen in fig. 2,) nearly restored to its natural degree of bending at the ankle. The angle formed by the foot-piece (*b b*) of the apparatus with the leg-piece (*a a a*) can be increased or diminished by turning the windlass (*c*) forwards or backwards, and by that means loosening or tightening the cord (*k k*), which, after passing through the holes (*l l*) of the foot-piece (*b b*), and over the pulleys (*e e*), is fastened to the windlass. The foot-piece of the apparatus can be rendered oblique by turning the thrumb-screw and loosening the slide (*f*) through which the axle (*g*) passes. The spring (*h*), which is received between the teeth of the cog-wheel (*d*), prevents the windlass from turning backwards, except when the surgeon purposely raises it. The double row of fissures (*i i i i i*) are for the passage of the straps (*m m o m m*) by which the foot is secured in the desired position; *p p* marks a cushion placed between the leg and the apparatus.

The second apparatus serves for the treatment of a greater variety of cases, either of T. equinus or T. varus, whether or no the operation of dividing any tendon be deemed requisite. It has, moreover, the advantage of enabling the patient to walk with less difficulty during the process of cure." 35.

FIG. XIV. represents the second apparatus mentioned in the text, made for a left foot. The utility of this apparatus in the cure of T. varus exceeds that of any other instrument which I have resorted to or found in use by others. Its superiority depends upon the application of an elastic power for the purpose of overcoming the resistance of the adductor muscles, the internal ligaments of the ankle, and the contracted tissues on the inside of the foot, instead of relying solely upon the inelastic pressure of screws and straps. The elastic property of steel springs, by the constancy of their action, approaches somewhat to the nature of the contractility of animal tissues, and is capable of effecting the elongation of contracted muscles or ligaments with more readiness and less tightness of the apparatus than a greater amount of the pressure of screws, straps, and bandages, which, in opposition to the elastic pressure or traction of the spring, may be called *dead* pressure. This apparatus is calculated to overcome the threefold alteration of the position of the foot which characterises Talipes varus; and by substituting an unpright firm bar of steel for the spring *c*, is well adapted to cure Talipes equinus, whether or no any operation be necessary.

The letters *a a* and *b* designate a shoe formed of iron, covered with leather, and well padded with horse-hair; *c* is a steel spring, which, by means of the padded strap *e*, is worn upon the outside of the leg; *i* and *f* shew a strap and bandage to go round the ankle, by which the heel is held securely in the shoe. The straps *g g* hold the heel firmly towards the inside of the shoe, whilst the short spring *d* draws the toes and front of the foot outwards, by means of the padded strap *h* passed round it. The screw *l*, which moves through a female screw *k*, (itself fixed as a rotatory rivet to the spring *c*,) acts upon the ankle-joint, and enables the surgeon to bend it as rapidly or as gradually as he may please. In cases where the tendo Achillis has been divided, the

After the healing of the wound, Dr. Little recommends great caution in the mode of applying the extension, &c. The skin should be protected from friction and uneven pressure by the application of an elastic cotton-web bandage, and by filling up with wadding those inequalities of the surface of the tarsus which arise, in many instances, from unequal projection of the bones.

In many cases, says our author, the slightest pressure exercised by the instrument will suffice to overcome the deformity without producing pain, provided attention be daily paid, and the straps and screws be tightened whenever they become loosened by the progress which the foot makes in the required direction. In other subjects, where the resistance is greater, more pressure is required, accompanied by a greater degree of pain; but the surgeon must not always expect to restore the foot rapidly to its natural position, nor must he proceed with any thing but gentleness and caution. He must avoid, so far as can possibly be done, the production of excoriations, sloughing, &c. which might seriously retard or altogether prevent the success of the operation.

"Occasionally the pain in the sole of the foot, produced by the pressure requisite to bend the ankle-joint, is intolerable: I can well appreciate the amount of suffering thus occasioned, having experienced it to a great degree in my own case. After witnessing in many patients the distressing pain felt in the sole of the foot when the form of the bones, or corns, caused particular parts of the sole to be disproportionately compressed, about two years since, at the suggestion of Mr. Robert Davey, of Great Ormond Street, I placed an air-cushion between the sole of the foot and the foot-piece of the apparatus, in order to produce an equal distribution of the pressure over the whole of the sole. The pain was instantaneously relieved; and I am indebted to the use of the air-cushion for the comparatively little pain with which the cure of numerous cases has been attended." 36.

Of course, particular cases will demand particular modifications of the use of a screw for bending the ankle-joint is less objectionable than any similar inelastic power would be were the tendon intact. The greater part of the resistance to *bending* the foot is removed by that operation; but as the active causes of the *adduction* and the *rotation inwards* of the foot, analogous to supination of the hand, remain, the springs, by being braced up against the foot and leg more or less tightly, according to the exigencies of the case, tend constantly to regain the form which they have when unshackled by the straps, and thus gradually overcome the contracted muscles and ligaments, and draw the deformed foot in two directions opposed to the deformity—those of abduction and rotation outwards. If the surgeon, acquainted with the anatomy and pathology of Talipes varus, will put his sound foot into one of these instruments, and endeavour whilst his foot is there to give it the form of T. varus, he will feel how strongly the springs oppose his attempts; in fact, he will be convinced that if he wore the instrument sufficiently long, his sound foot would be converted into a deformity resembling the opposite of T. varus, namely, Talipes valgus. It is at the point intermediate between these two diseases that the practitioner must stop in the treatment of the former—at the position of the foot intermediate between adduction and abduction—at the point corresponding to the natural position.

apparatus. In children, the treatment may be interrupted by the occurrence of the maladies to which they are particularly prone. It will be an object not to lose the ground that has been gained.

The apparatus which Dr. Little has used for the treatment of Talipes valgus is similar to the second instrument described for the cure of T. varus (fig. 14,) with this difference only, namely, that the springs, which in the latter case, are placed upon the outer side of the limb, are required in T. valgus to be worn upon the inner side.

Dr. Little takes care to point out that the extent of external deformity of the foot does not determine the length of time necessary to effect a cure; other circumstances exercise a much greater influence in this respect. One of the most important is the degree of alteration from the natural shape and position which the individual bones of the tarsus have undergone, produced either by the occurrence of the disease at an early period of the development of the embryo, when the bones were yet soft, by the wearing improper instruments, or walking with the foot in its deformed shape, and thus injuring the bones by attrition against the earth. The shortened condition of some of the ligaments of the ankle in T. varus and equinus, particularly the ligamentum deltoideum, and the ligaments of the under surface of the tarsal arch, are serious difficulties to be overcome in long-standing cases. Those muscles of the sole of the foot which it is inexpedient to divide, and which, in some cases of T. varus and equinus, draw the anterior extremities of the metatarsal bones backwards (towards the posterior tuberosity of the os calcis,) for a lengthened period resist our endeavours to stretch them.

Dr. Little admits that there are some cases of T. varus and equinus where, after division of the tendo Achillis and healing of the puncture, the foot might be at once restored to its natural position; but to avoid any interference with the reunion of the tendon, he prefers the gradual replacement of the foot. And he adds:—

"In the average of cases of club-foot, whether congenital or otherwise, two or three weeks elapse after the operation before sufficient bending of the joint is obtained; previously to which, however, the patient is allowed to walk, wearing the apparatus: after the expiration of another fortnight, the greater part of the tenderness of the ankle-joint, produced by the stretching of the ligaments, subsides. By this time the union of the tendon has acquired sufficient firmness to allow of walking and exercise in the open air. In the greater number of cases, particularly in children, the ankle-joint requires to be supported by a modification of the instrument used to effect the extension, or by one or two thin steel springs, or pieces of whalebone, sewn between the outer leather and the lining of the side of a lace-up boot. Some time elapses before the gait of the individual acquires the ultimate perfection of which it is susceptible. Children advance more slowly in this respect than adults, as they limp if they feel the least uneasiness, and cannot take so much pains with their manner of walking. I have had many adult patients who, within two months after the performance of the operation, have walked several miles, and have had but little appearance of lameness. I have, however, treated one very bad case of adult congenital T. varus, which was under my care for nine months,—that of a gentleman who had walked from infancy without the slightest mechanical support, and whose foot had consequently reached a frightful stage of deformity; but the ultimate success, notwithstanding the attendant difficulties, enables me to refer to this case as a triumphant proof of the advantage of the Stromeyerian method of cure." 39.

We shall here take leave of our author and the subject. We quite agree with him that the operation of Stromeyer, and its modifications, constitute a very important improvement in the practice of surgery. The deformities that they relieve are numerous and afflicting—the relief that they afford considerable—and operations of this character, are, perhaps, a greater boon to humanity than some of greater pretension and of less extensive application.

It only remains for us to express our good opinion of the manner in which Dr. Little has performed his task. His book will prove a very useful one, and we recommend it in strong terms to the profession.

## Figures and Tables

**1. f1:**
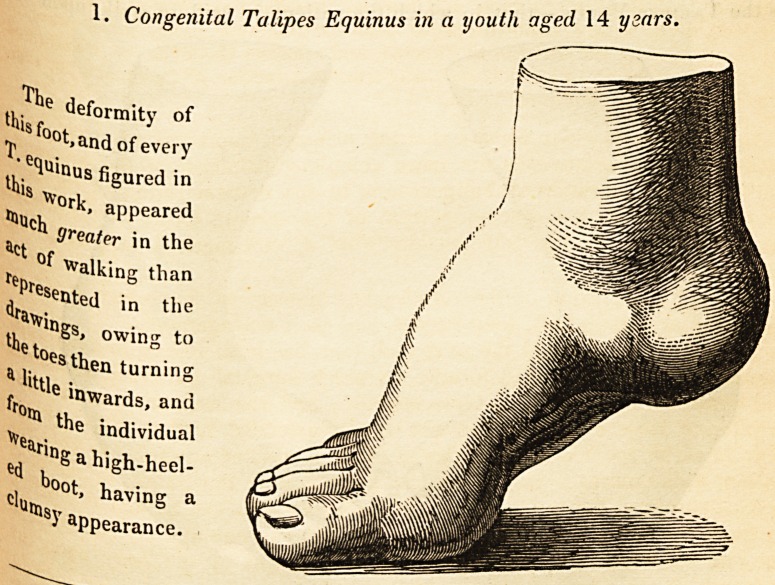


**2. f2:**
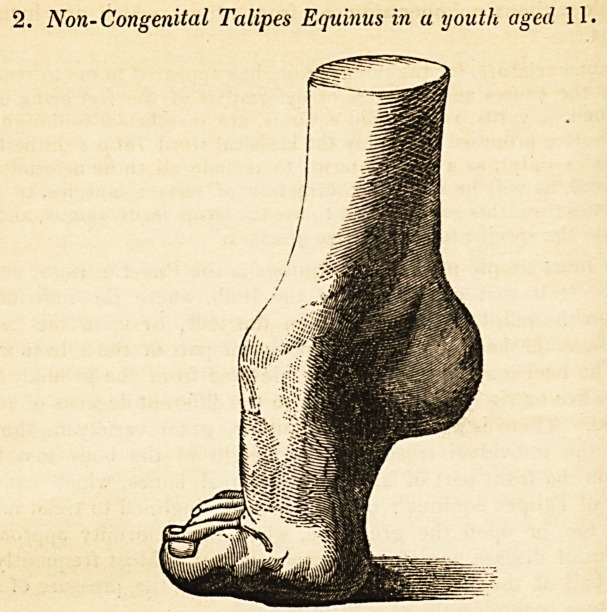


**Fig. 3. f3:**
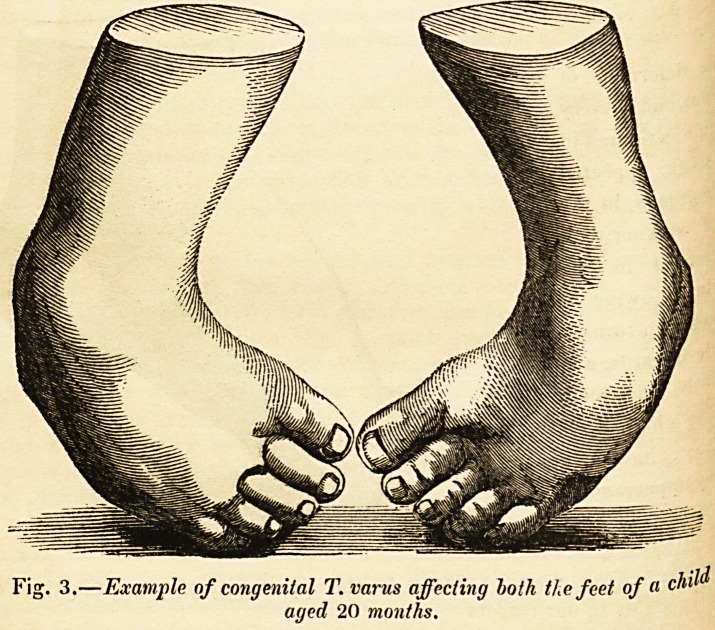


**Fig. V. f4:**
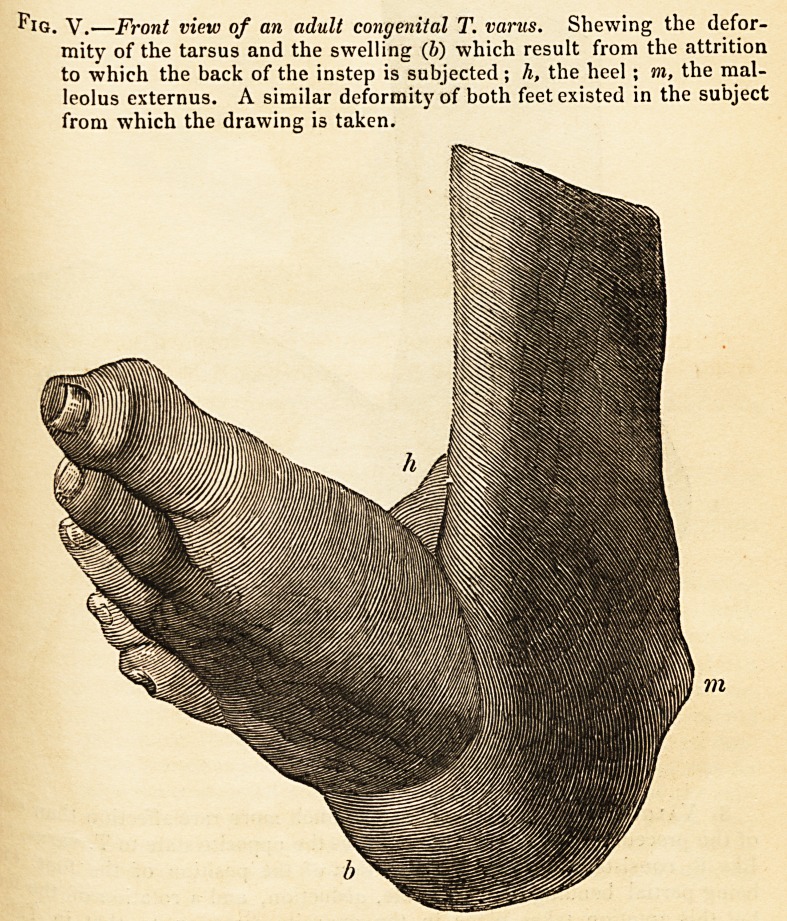


**Fig. VI. f5:**
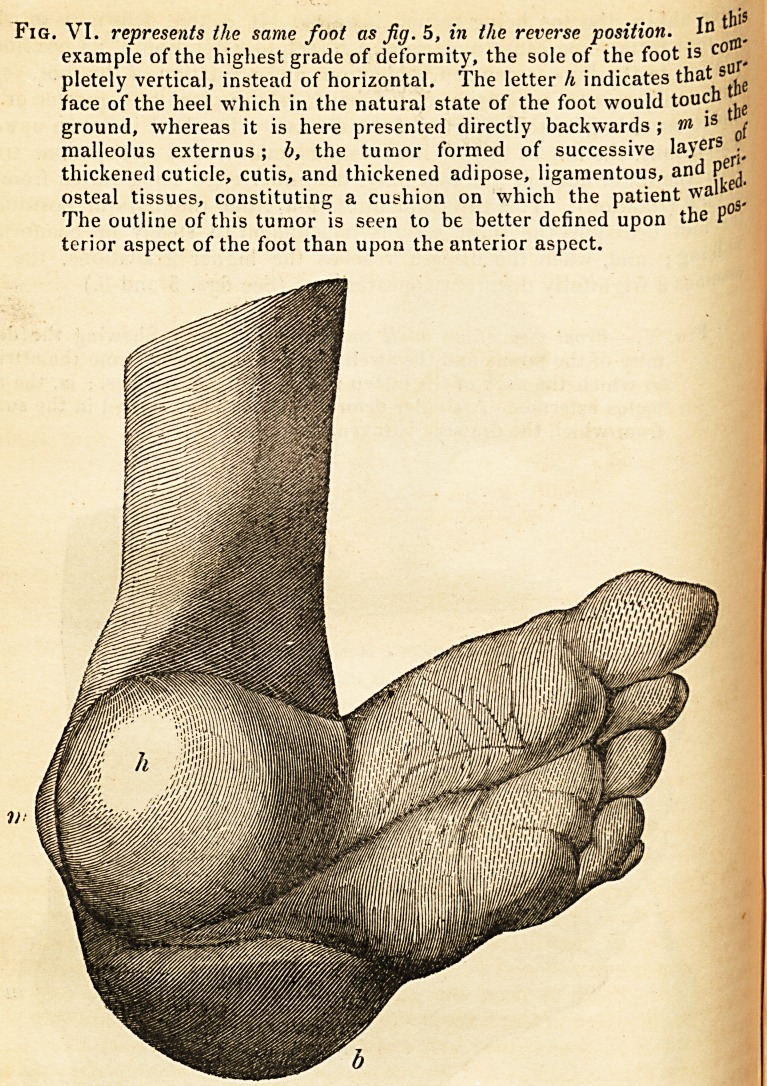


**Fig. IV. f6:**
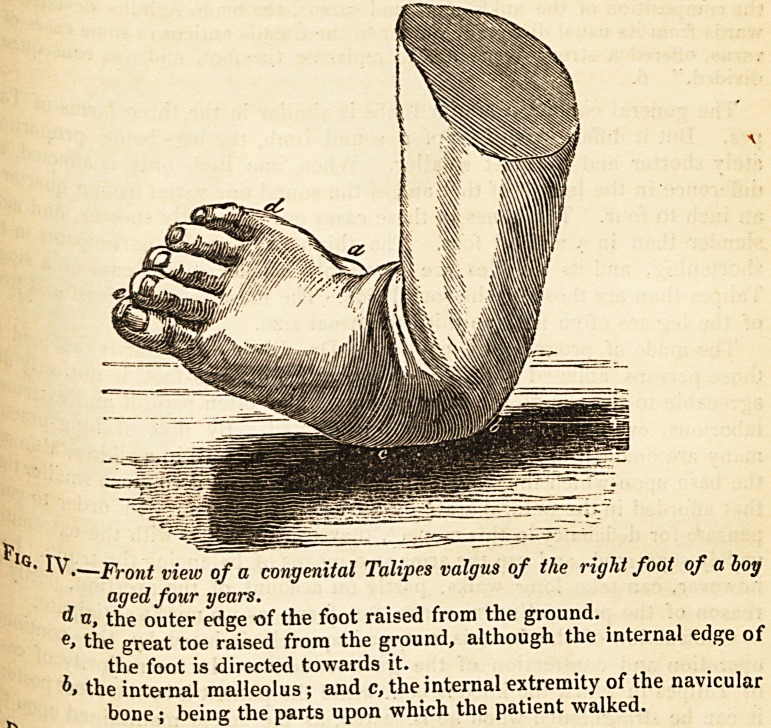


**Figure f7:**
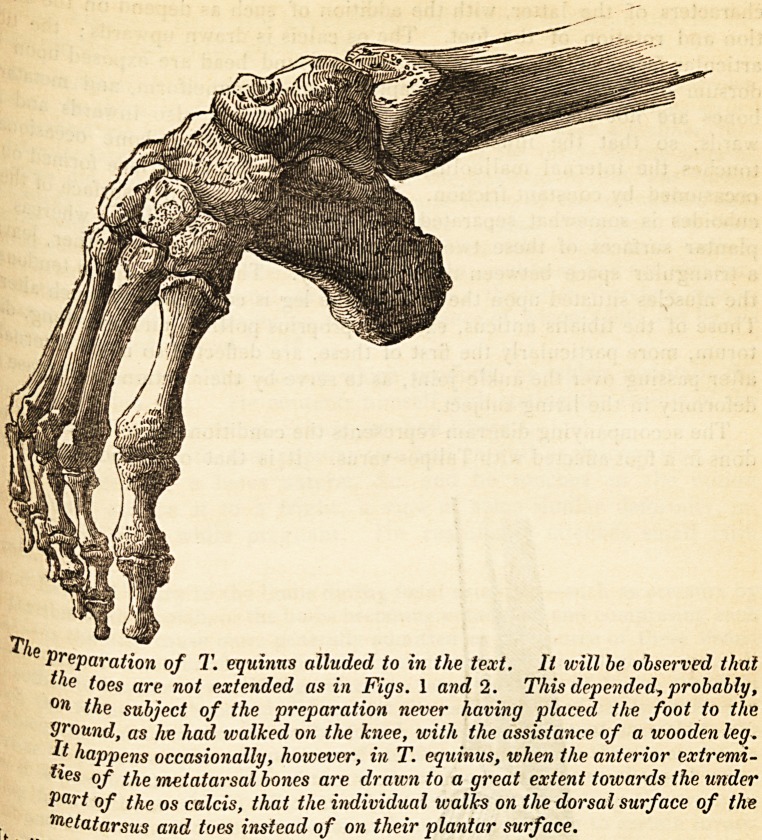


**Figure f8:**
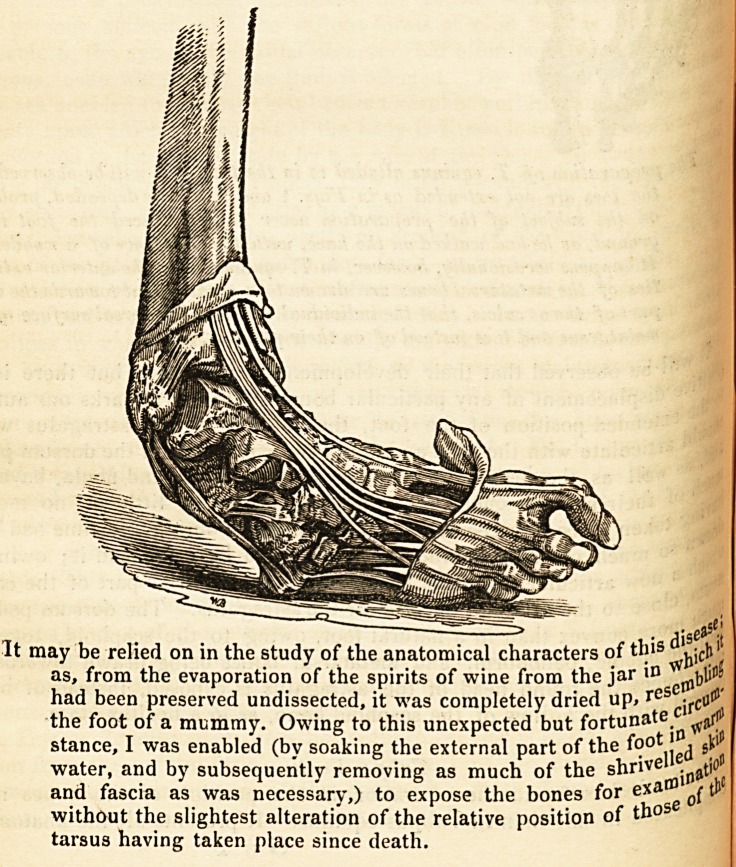


**Fig. 39. f9:**
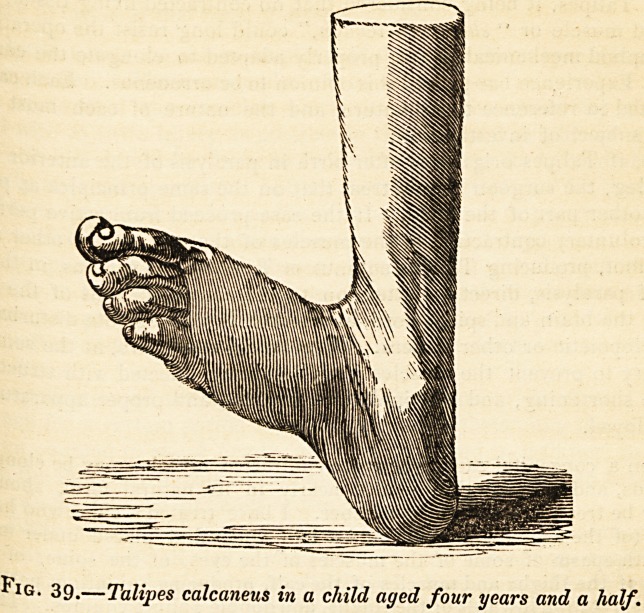


**Fig. XIII. f10:**
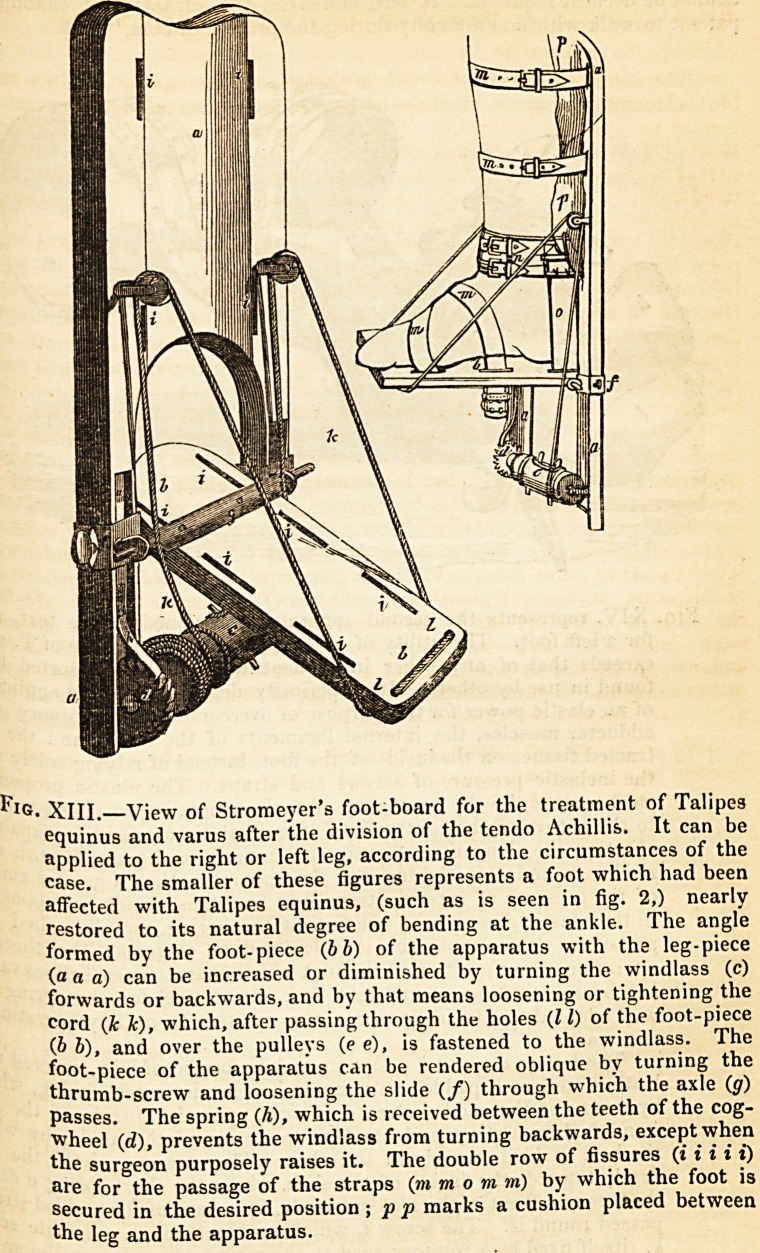


**Fig. XIV. f11:**